# Lung ultrasound to predict mechanical ventilation weaning failure

**DOI:** 10.1186/2197-425X-3-S1-A317

**Published:** 2015-10-01

**Authors:** LY Delgado Ayala, O Torres, A Sanchez-Calzada, JL Navarro, A Torres, R Gastelum, P Romano Albornoz, M Arcos, EE Monares, J Aguirre, J Franco

**Affiliations:** ABC Medical Center, Intensive Care, Mexico City, Mexico; ABC Medical Center, Orthopedics, Mexico City, Mexico

## Introduction

Mechanical ventilation weaning failure accounts for the 10-20% of all planed cases despite having indexes and spontaneous test which increases morbidity and mortality.

## Objectives

To evaluate the usefulness of lung ultrasound to predict mechanical ventilation weaning failure.

## Methods

Prospective observational study completed from April 2013 to April 2015. Including adult patients with mechanical ventilation form more than 24 hours that met the international consensus conference criteria. Patients with less than 18 years old or those with the order of limiting therapeutic effort. Discontinuation from mechanical ventilation was attempted at clinical judgment of the medical team at charge, according to the consensus criteria. Pressure support ventilation were started at 7 cmH2O, CPAP 0, Fi02: 0.35% for 30 minutes and indexes (IWY, tracheal airway occlusion pressure P01, VRS Delta VRS, Sv02, NIF, time to reach NIF,CV, p01 x VRS from minute 30) were calculated at minute 1 and 30. Lung ultrasound was performed. It was categorized in patterns A, B o AB. Group A: successful extubation and Group B: extubation failure.

For quantitative variables T-test and U-Mann Whitney tests were performed. Crosstabs and Chi square test were performed to compare differences in qualitative variables. ROC were performed.

## Results

A total of 79 patients were analyzed. With 42 (53.2%) male patients and mean age of 64 ± 16.23 years with a maximum age of 88 years. A total of 17(21.5%) of the population presented extubation failure.

Significant difference in length of stay were founded with 13 ± 14.39 days in the A group and 27 ± 23.68 days in the B group. P01 values at minute 1 showed significant difference with a mean value of -2.19 ± in the A group and -3.6 in the B group.

ROC curves analysis showed that non of the indexes (IWY, VRS deltha, VRS, Sv02) had significant value except for the lung ultrasound at minute 1 and 30. A B pattern at minute 1 showed an area under the curve (AUC) of 0.7 (p = 0.031, CI 0.54-0.85) with a sensibility of 81% and specificity of 60% for predicting weaning failure. Also a B pattern at minute 30 showed an AUC of 0.68 (P= 0.046, CI 0.52-0.85)) with a sensibility of 80% and specificity of 50% for predicting weaning failure.

When analyzing only the group of patients who presented B patterns at minute 1 ROC curves showed significant value for predicting extubation success with a cutoff point of ≥-2.35cmH2O for P01 at minute 1 (AUC 0.86, CI 0-1.00, p = 0.016, sensibility 80%, specificity 80%). And the subcategory of B pattern at minute 30 a cutoff value of ≥-2.45cmH2O of P01 at minute 30 also showed significant value for predicting extubation success (AUC 0.82, CI 0.64-1.00, p = 0.42,

## Conclusions

Lung ultrasound combined with P01 at minute 1 and 30 respectively is a promising index to predict extubation success.and needs further validation.

